# Supplementary figures and images for: Absolute concentration estimation of COVID-19 convalescent and post-vaccination IgG antibodies
Source: PLoS One. 2024 Nov 1;19(11):e0311777. doi: 10.1371/journal.pone.0311777 (PMC11530011; doi:10.1371/journal.pone.0311777)

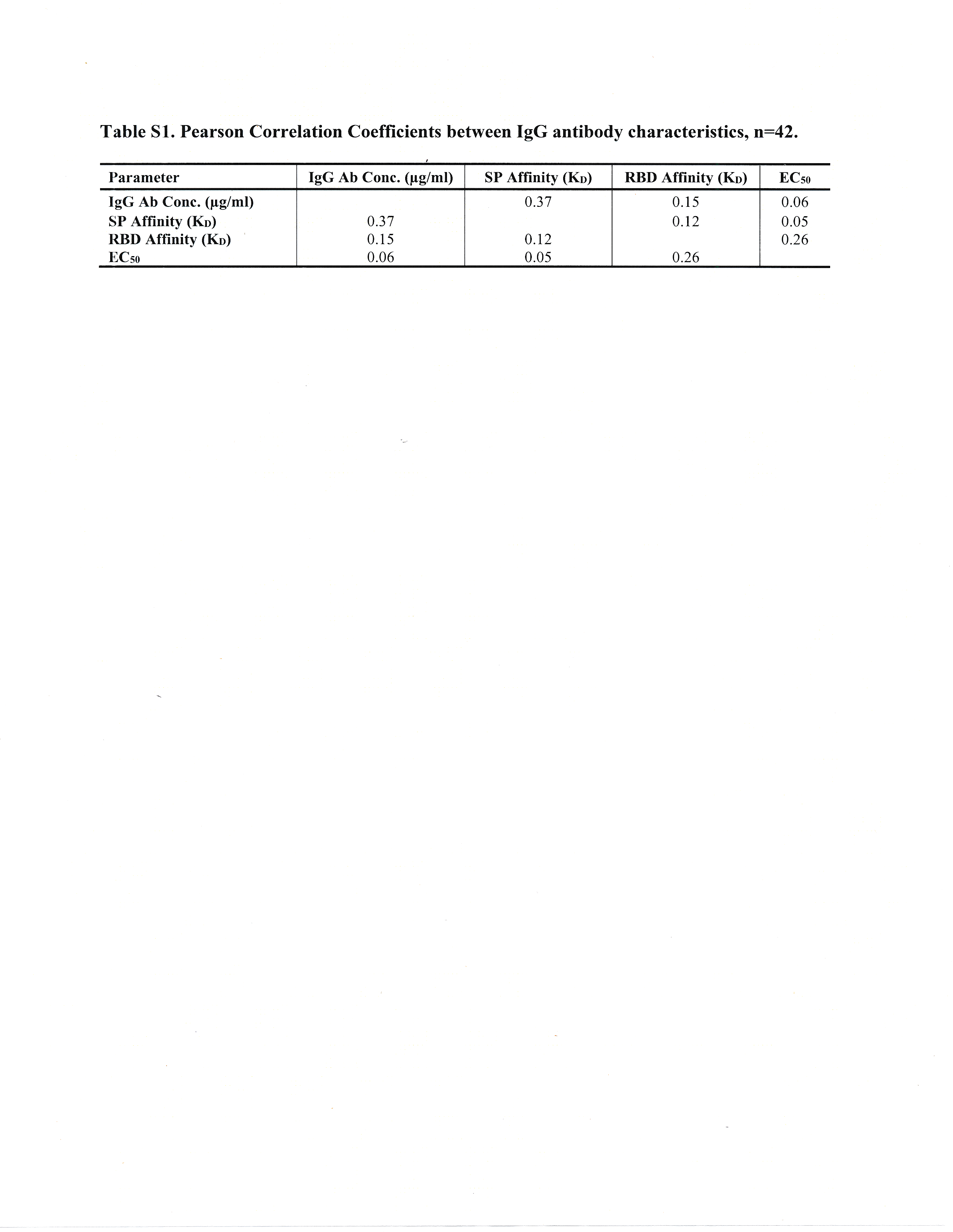

Supplement: S1 Table — (TIF) [file pone.0311777.s001.tif]

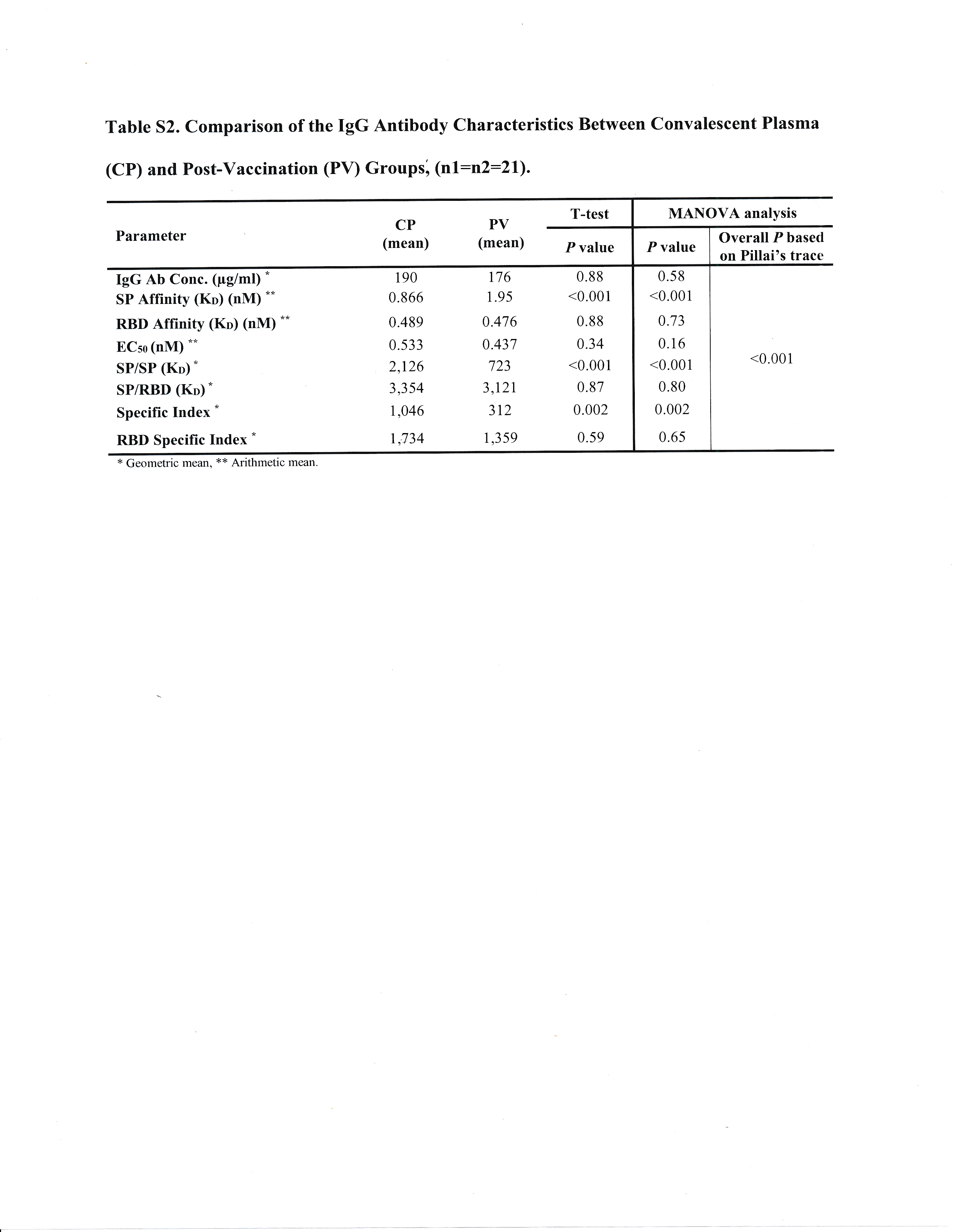

Supplement: S2 Table — (TIF) [file pone.0311777.s002.tif]

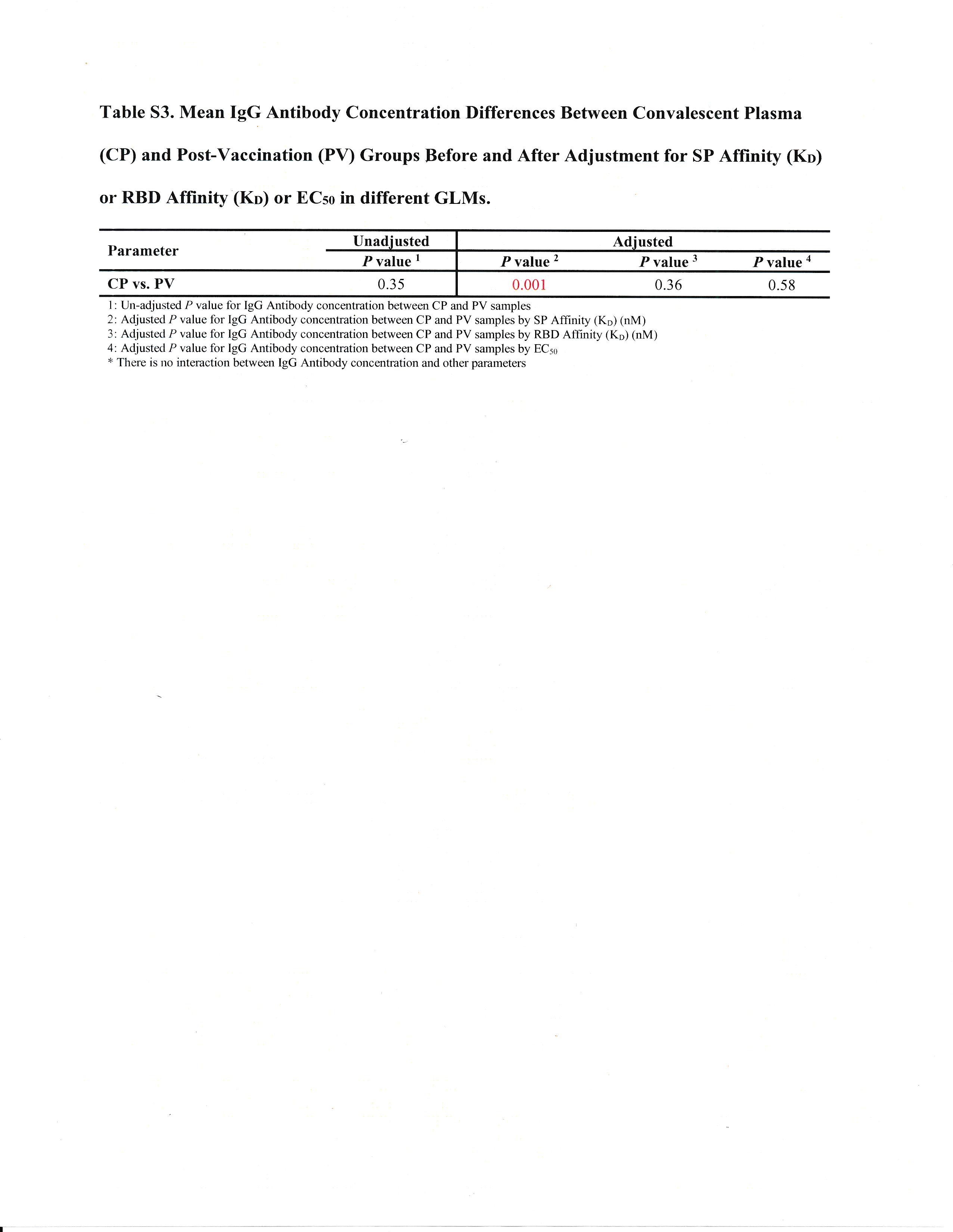

Supplement: S3 Table — (TIF) [file pone.0311777.s003.tif]

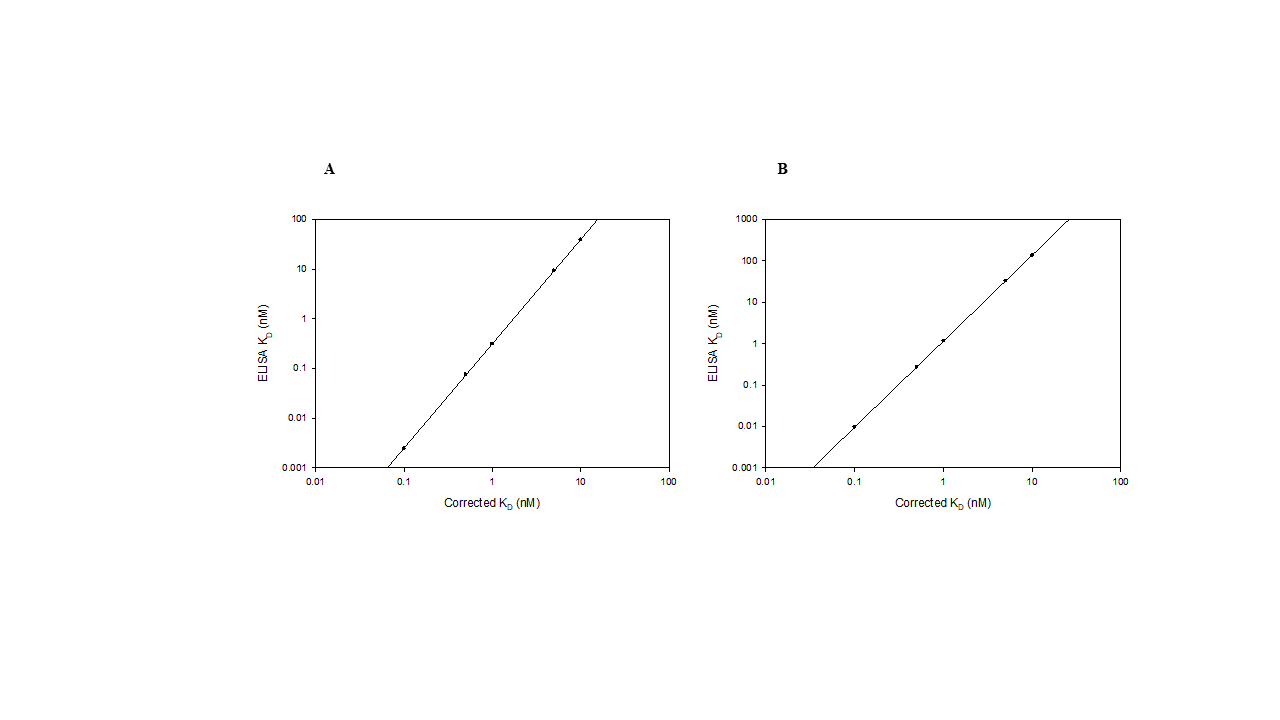

Supplement: S1 Fig — (TIF) [file pone.0311777.s004.tif]

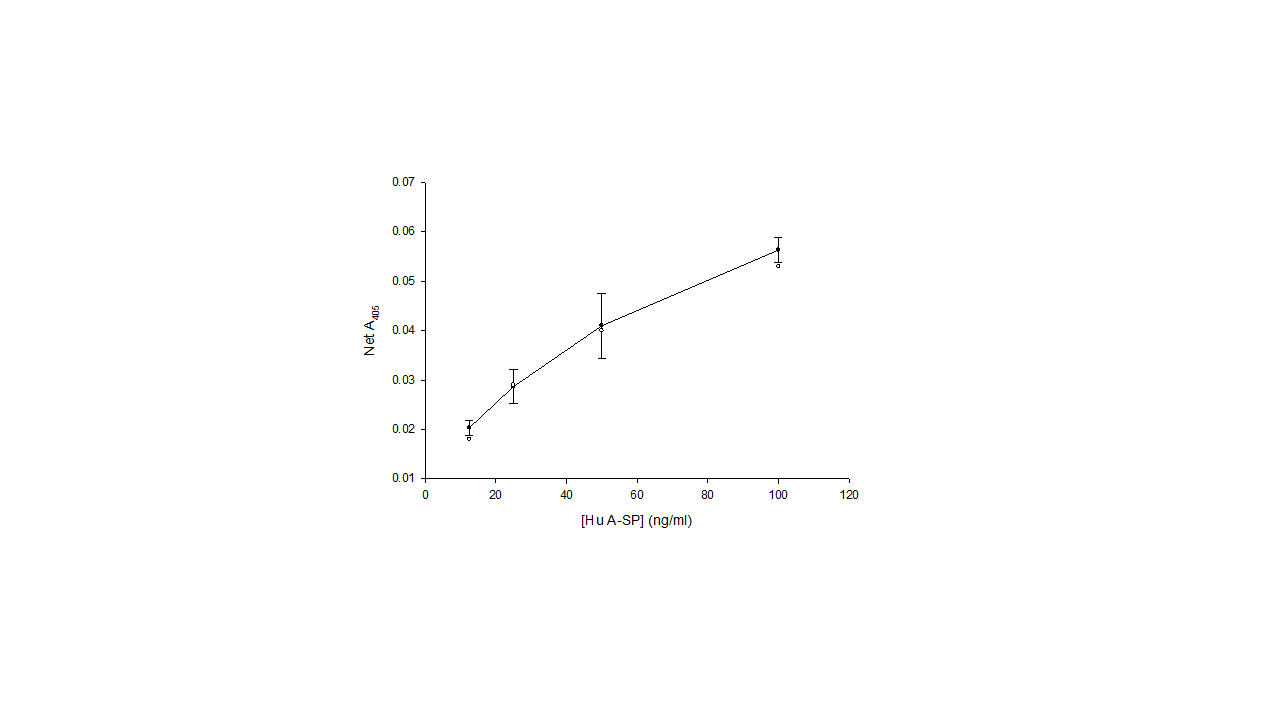

Supplement: S2 Fig — (TIF) [file pone.0311777.s005.tif]

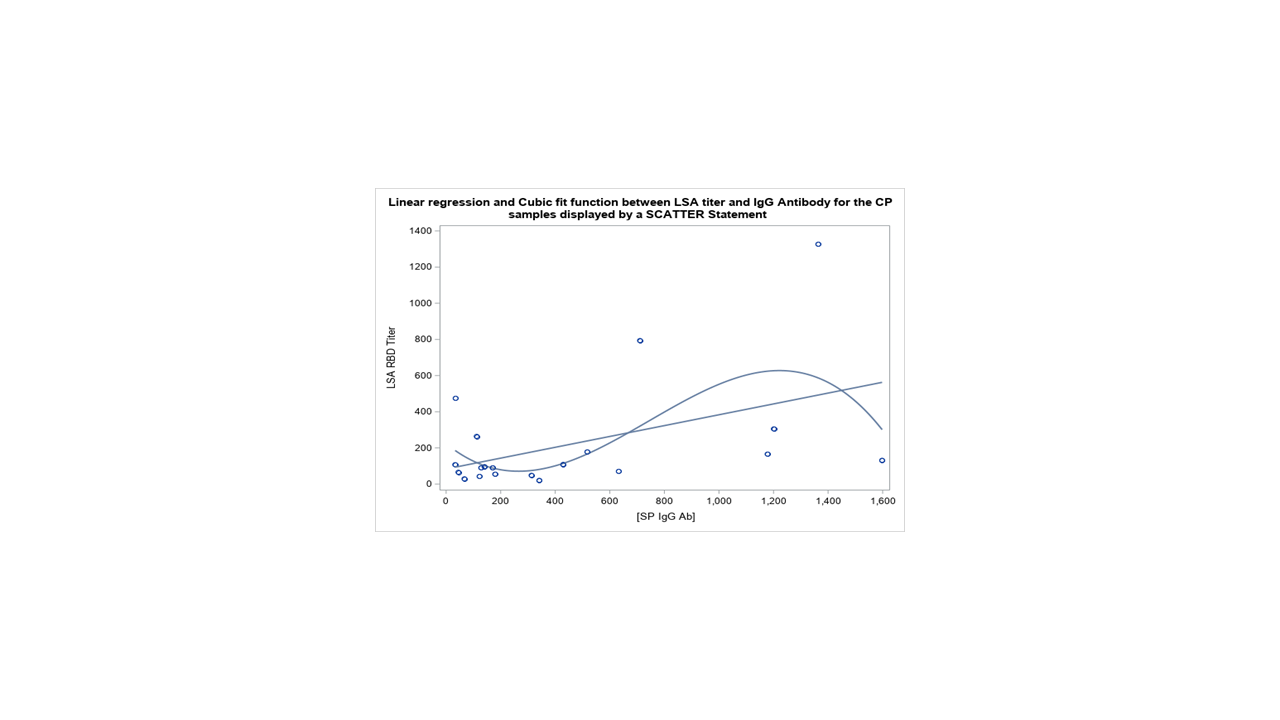

Supplement: S3 Fig — (TIF) [file pone.0311777.s006.tif]
