# Supplementary material for: Absolute concentration estimation of COVID-19 convalescent and post-vaccination IgG antibodies
Source: PLoS One. 2024 Nov 1;19(11):e0311777. doi: 10.1371/journal.pone.0311777 (PMC11530011; doi:10.1371/journal.pone.0311777)
Supplement: Attachment — Submitted filename: Klegerman Response to Reviewers.docx [file pone.0311777.s007.docx]

**PONE-D-24-05130**

**Response to Reviewer Comments**

We would like to thank the reviewer for helpful comments that have improved the quality of the manuscript. We have amended it in accordance with the comments, as follows.

1. **The authors should discuss the findings of the current study in a bit more detail. The discussion portion of the article focuses more on the findings of other research articles and does not discuss the data submitted in this article enough.**

We have added three paragraphs to the Discussion discussing the justification for the study design and methodology, evidence for validity of the results, and advantages of the approach.

**2. The authors also need to discuss the future implications of the current study and how it can be useful for any future research endeavors in the field.**

We have added several sentences at the end of the manuscript describing specific studies that can be performed based on ability to measure actual antibody concentrations.
